# Incidental germline findings during molecular profiling of tumor tissues for precision oncology: molecular survey and methodological obstacles

**DOI:** 10.1186/s12967-022-03230-z

**Published:** 2022-01-15

**Authors:** Alexandra Lebedeva, Yulia Shaykhutdinova, Daria Seriak, Ekaterina Ignatova, Ekaterina Rozhavskaya, Divyasphoorthi Vardhan, Sofia Manicka, Margarita Sharova, Tatiana Grigoreva, Ancha Baranova, Vladislav Mileyko, Maxim Ivanov

**Affiliations:** 1 Atlas Oncodiagnostics, LLC, Moscow, Russia; 2 Sechenov University, Moscow, Russia; 3 Salem Hospital, Salem, MA USA; 4 Department of chemotherapy №2, Federal State Budgetary Institution «N.N. Blokhin National Medical Research Center of Oncology» of the Ministry of Health of the Russian Federation, Moscow, Russia; 5 Department of Oncogenetics, Institute of Higher and Additional Professional Education, Research Centre for Medical Genetics, Moscow, Russia; 6 School of Systems Biology, George Mason University, Mannas, VA USA; 7 Moscow Institute of Physics and Technology, Dolgoprudny, Moscow Region Russia

## Abstract

**Background:**

A fraction of patients referred for complex molecular profiling of biopsied tumors may harbor germline variants in genes associated with the development of hereditary cancer syndromes (HCS). Neither the bioinformatic analysis nor the reporting of such incidental germline findings are standardized.

**Methods:**

Data from Next-Generation Sequencing (NGS) of biopsied tumor samples referred for complex molecular profiling were analyzed for germline variants in HCS-associated genes. Analysis of variant origin was performed employing bioinformatic algorithms followed by manual curation. When possible, the origin of the variant was validated by Sanger sequencing of the sample of normal tissue. The variants’ pathogenicity was assessed according to ACMG/AMP.

**Results:**

Tumors were sampled from 183 patients (Males: 75 [41.0%]; Females: 108 [59.0%]; mean [SD] age, 57.7 [13.3] years) and analysed by targeted NGS. The most common tumor types were colorectal (19%), pancreatic (13%), and lung cancer (10%). A total of 56 sequence variants in genes associated with HCS were detected in 40 patients. Of them, 17 variants found in 14 patients were predicted to be of germline origin, with 6 variants interpreted as pathogenic (PV) or likely pathogenic (LPV), and 9 as variants of uncertain significance (VUS). For the 41 out of 42 (97%) missense variants in HCS-associated genes, the results of computational prediction of variant origin were concordant with that of experimental examination. We estimate that Sanger sequencing of a sample of normal tissue would be required for ~ 1–7% of the total assessed cases with PV or LPV, when necessity to follow with genetic counselling referral in ~ 2–15% of total assessed cases (PV, LPV or VUS found in HCS genes).

**Conclusion:**

Incidental findings of pathogenic germline variants are common in data from cancer patients referred for complex molecular profiling. We propose an algorithm for the management of patients with newly detected variants in genes associated with HCS.

## Introduction

NGS is gaining recognition as an in vitro companion diagnostic aid in clinical decision-making. In 2017, Oncomine DX Target Test became the first NGS-based test approved by the FDA for a set of non-small-cell lung cancer-related genetic alterations [1, 2]. This was followed shortly by FoundationOne CDx [3, 4], FoundationFocus CDxBRCA [5], and MyChoice HRD CDx [6]. In the context of managing oncology patients, NGS is predominantly used as a tool for predicting the efficacy of therapies that may be influenced by the presence or lack of specific somatic mutations [7].

Compared to conventional methods for DNA analysis, such as Sanger sequencing or PCR (Polymerase Chain Reaction), NGS can identify a large array of DNA regions, which are not limited to the short list of mutations that clinicians expect to find in a patient with a certain diagnosis. Hence, a typical result of diagnostic NGS is represented by a list of identified mutations, only some of which are related to the specific disease phenotype, and others, unrelated to the specific disease, that have the potential for clinical relevance [8]. The latter type of reported variance is known as “incidental”, or secondary findings. Incidental findings of germline origin are especially important for both managing the patients’ health and correctly assessing the risks of their relatives developing pathologies [9, 10].

The reporting of germline findings usually follows the ACMG/AMP guidelines or their refined version, the SHERLOC guidelines. These guidelines propose that each sequence variant should be assessed according to a 5-Tier system based on objective criteria, such as population frequency of a genetic alteration, computational predictions of pathogenicity, or existing research on the functional effect of a genetic variant. However, existing guidelines are not disease-specific, and are mostly suitable for hereditary diseases associated with highly penetrant genes. Next, the criteria in the ACMG/AMP or SHERLOC guidelines do not include the medical history of the proband in the decision-making process. Another set of existing guidelines, NCCN, aids in assessing familial oncological risks by focusing mostly on specific, highly penetrant cancer-susceptibility genes such as BRCA1/2, PTEN, or TP53 [11]. Therefore, no existing guidelines are suited for interpreting NGS data obtained from oncology patients assessed by general oncology practices.

Here, we report our first-hand experience with NGS analysis of a large population of cancer patients. We present the statistics on identified genetic alterations and their interpretations, along with a detailed dissection of methodological obstacles faced in course of the identification of such incidental findings.

## Methods

### Sample collection and sequencing

Tumor samples were presented by FFPE tissue blocks from each patient. Tumor genomic DNA was extracted from 4 to 8 freshly cut sections of FFPE tissue using GeneRead DNA FFPE kit (Qiagen) according to the manufacturer's protocol, including the step of specific removal of deaminated cytosine residues by the enzyme Uracil-N-Glycosylase (UNG). The concentration of the DNA was determined using the Qubit dsDNA HS Assay Kit. DNA quality was evaluated by the PCR-based QuantumDNA kit (Evrogen).

Depending on the panels used, 409 or 411 genes were analysed. Target region amplification was performed employing two panels: Ion AmpliSeq Comprehensive Cancer Panel (Thermo Fisher Scientific Inc.) and the Atlas ABC panel. The Atlas ABC panel was designed via Ion Ampliseq Designer (Thermo Fisher Scientific Inc.) through the White Glove process and includes two primer pools, comprising 409 amplicons within 4 cancer-related genes: BRCA1, BRCA2, ATM, and CHEK2. Ion AmpliSeq™ Comprehensive Cancer Panel (CCP) targeted 409 genes and 15,992 amplicons in four pools. Five nanograms of FFPE-derived (tumor) DNA were used to prepare sequencing libraries using the Ion Ampliseq library preparation kit v2.0 and The Ion Torrent Dual Barcode Kit 1-96 (Thermo Fisher Scientific Inc) according to the manufacturer's protocol. The quality and quantity of the barcoded libraries were determined using gel electrophoresis and Qubit 2.0 Fluorometer TM (Thermo Fisher Scientific Inc). Pooled libraries were combined and diluted to 10 pM and templated on the Ion Chef and loaded onto an Ion 540 chip. The Ion 540 chip was sequenced on the Ion GeneStudio S5 System (Thermo Fisher Scientific).

### Data analysis and interpretation

Raw sequence data analysis, including base calling and demultiplexing, was performed using the Torrent Suite Software v.4.0.2 (Thermo Fisher Scientific, Inc.) Sequenced reads were mapped using the human genome as a reference (version GRCh37.p13), employing Burrows-Wheeler Aligner (BWA-mem, version 0.7.7-r441) or software from the sequencing platform provider. Software from sequencing platform provider (Ion Torrent Variant Caller version 5.8-18) was used to call somatic SNVs and small InDels. Detected variants were classified as hotspot and non-hotspot based on prevalence in the COSMIC database (COSMIC count of 10 was used as the threshold) [12]. Filtering methods were different for candidate variations in positions of recurrent mutagenesis and all the others. Thresholds used for hotspot variant filtering were the following: coverage depth > 19; number of mutant reads > 7; variant allele frequency > 2%. Thresholds used for non-hotspot variant filtering were the following: coverage depth > 19; number of mutant reads > 9; variant allele frequency > 5%. Stand bias analysis was performed employing in-house scripts. Analysis of CNVs was performed using ONCOCNV software [13]. Minor-allele frequency data were referenced using the 1000 Genomes Project Database [14], the NHLBI GO Exome Sequencing Project [15], and the TOPMED project [16]. Further analysis was focused only on variants in genes potentially associated with the development of hereditary cancer syndromes: BRCA1, BRCA2, MLH1, MSH2, MSH6, PMS2, EPCAM, APC, MUTYH, CDKN2A, CDK4, TP53, PTEN, STK11, CDH1, BMPR1A, SMAD4, PALB2, CHEK2, ATM, NBN, BARD1, BRIP1, RAD51C, RAD51D, POLD1, POLE, GREM1, HOXB13, AXIN2, GALNT12, RPS20, RNF43, NTHL1, MSH3, SMARCB1, and BLM.

### Discrimination of variants on somatic and germline

#### ISOWN

The discrimination between somatic and likely-germline missense mutations was performed employing ISOWN [17] with further manual curation and manual tools. In summary, ISOWN is a machine-learning-based method designed to predict whether a certain variant is germline or somatic based on several factors, including population frequency, variant allele frequency (VAF), VAF of the adjacent polymorphisms, nucleotide composition, potential damaging effects, and presentation of a variant in databases. ISOWN classified each missense variant as germline or somatic. Since ISOWN is only intended for the discrimination of single nucleotide variants, small deletions and insertions were considered to be somatic or germline based solely on manual curation.

#### Principles of manual curation

To take into account additional factors that were not otherwise considered by ISOWN, manual curation was performed for all of the detected variants. While manually determining the origin of the detected variants, the following was considered: (1) VAF of passenger variants are generally lower than VAF of driver mutations [18]; (2) the likelihood of detecting pathogenic variants in genes potentially associated with the development of HCS (or other mendelian diseases) is lower in patients who do not have an overt hereditary disease according to the clinical diagnosis (including personal and family history of cancer, morphological and histological presentation of the disease); (3) if no CNVs are detected in locus, the chances of detecting a variant with VAF lower than tumor cellularity are low, and (4) the patterns of germline and somatic mutations in certain genes were taken into account [19–21]. As a result of manual curation, variants were classified as germline heterozygous, germline homozygous, somatic, or of uncertain origin.

#### Variant interpretation

Patient tumor samples were analyzed to identify germline variants, potential associations with hereditary cancer syndromes, as well as potential predictive and prognostic biomarkers. Clinical interpretation of detected variants was performed to identify their potential association with hereditary cancer syndromes and aimed at classifying variants as pathogenic, likely pathogenic, variants of uncertain significance (VUS), likely benign, or benign. Clinical significance of individual variants in BRCA1 and BRCA2 genes was estimated using the ENIGMA database [22], while variants in APC, EPCAM, MUTYH, CDH1, GALNT12, MSH2, MSH6, PMS2, and MLH1 genes were assessed according to the InSiGHT database [23]. For variants with no estimated pathogenicity in these genes according to the aforementioned databases, as well as variants in other genes, we conducted a literature search for predicted impact on protein function as well as case–control and functional studies, according to ACMG guidelines [24]. These variants were then classified based on SHERLOC guidelines [25]. Only variants classified by manual curation as germline heterozygous or uncertain were subjected to clinical interpretation.

### DNA Sanger sequencing

Variants classified as uncertain by manual curation and pathogenic, likely pathogenic, or VUS based on clinical interpretation were validated by Sanger sequencing. Sanger sequencing was performed using the ABI PRISM BigDye Terminator Cycle Sequencing v.2.0 Ready Reaction kit and ABI PRISM 3730 DNA analyzer (Applied Biosystems) as previously described [26]. Blood samples were used for Sanger sequencing. All blood samples matched the corresponding tumor samples.

## Results

### Study population

From 07/2018 to 12/2019, 183 unselected adult patients satisfying eligibility criteria (see methodology) were referred for comprehensive molecular profiling at the discretion of their oncologists. In all 183 tumors, collected from the 23 tumor sites, including 34 colorectal, 24 pancreatic, 18 lung, 16 ovarian, 15 breast, 11 stomach, and others, DNA was extracted and NGS was performed. According to Oncotree classification [27], these tumors belong to 67 different histological and molecular tumor types. All patients were profiled on the Comprehensive Cancer Panel (Ion Torrent), covering a coding sequence of 409 oncogenes and tumor-suppressor genes. For 132 patients, additional sequencing was performed to include comprehensive coverage of BRCA1/2, ATM, and CHEK2 genes (Atlas ABC panel). Additional Sanger sequencing was performed for 7 patients (3.8%) with variants of likely germline origin and uncertain origin following manual validation to determine their somatic or germline origins The clinicopathological characteristics of the patients are shown in Table 1.

**Table 1:** The clinicopathological characteristics of the patients

Total	n	%
	183	
Age (years) at disease manifestation		
Median (IQR)	56 (44–65)	
< 40	14	7.7
40–49	19	10.4
50–59	21	11.5
60–69	23	12.6
70–79	11	6.0
≥ 80	2	1.0
Unknown	93	50.8
Sex		
Male	75	41.0
Female	108	59.0
Tumor site		
Colon and rectum	34	18.6
Pancreatic	24	13.1
Lung	18	9.8
Ovary/fallopian tube	16	8.7
Breast	15	8.2
Stomach	11	6.0
Cervix	8	4.4
Other, including unknown primary	8	4.4
Skin/melanoma	8	4.4
Soft tissue	7	3.8
Biliary	6	3.3
Head and neck	5	2.7
CNS/brain	4	2.2
Bladder/urinary	3	1.6
Uterus	3	1.6
Kidney	3	1.6
Bone	2	1.1
Small bowel	2	1.1
Prostate	2	1.1
Ampulla of Vater	1	0.5
Pleura	1	0.5
Testis	1	0.5
Liver	1	0.5
Stage		
I	7	3.8
II	15	8.2
III	12	6.6
IV	26	14.2
Not allowed to collect/not reported/unknown	123	67.2
Metastasis stage		
M0	65	35.5
M1	46	25.1
Not allowed to collect/not reported/MX/Unknown	72	39.3

### Sequencing results and variant origin discrimination

**Fig. 1:**
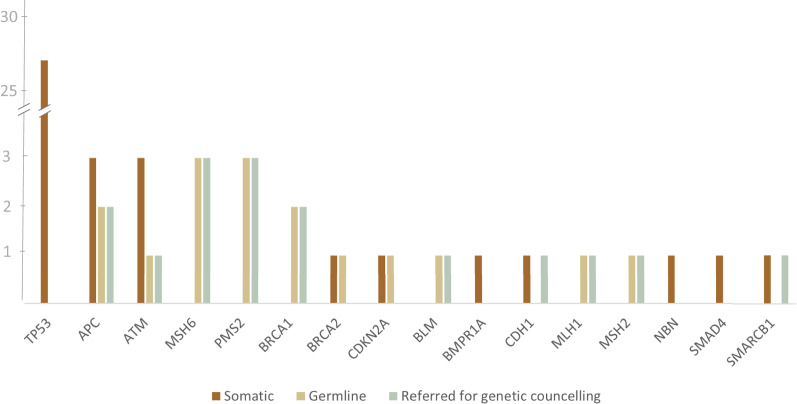
The distribution of somatic and germline variants by gene. Variants were classified as germline or somatic based on the results of manual validation and Sanger sequencing. The number of patients referred for genetic counselling is also shown

In total, from a sample of 183 patients, we detected 56 unique variants (Table 2, Fig. 1). Of those, 42 (75%) were missense, 9 (16%) were small insertions or deletions (indels), and 5 (9%) were nonsense mutations.

**Table 2:** Results of mutation detection by gene

Gene	Total variants	Origin status by manual curation		PV(LPV) (+VUS) across germline or uncertain origin
		Somatic	Germline (+variants of uncertain origin)	
TP53	27	27	0	0
APC	5	3	2	2
ATM	4	3	1	1
MSH6	3	0	3	3
PMS2	3	0	3	3
BRCA1	2	0	2	2
BRCA2	2	1	1	0 (+1)
CDKN2A	2	1	1	0
BLM	1	0	1	1
BMPR1A	1	1	0	0
CDH1	1	0	0 (+1*)	0
MLH1	1	0	1	1
MSH2	1	0	1	1
NBN	1	1	0	0
SMAD4	1	1	0	0
SMARCB1	1	0	1*	0
Total	56	38	17 (+1)	14 (+1)

*Based on the results of manual validation. However, based on the results of Sanger sequencing on the patients' normal tissue, these variants were found to be somatic, and the patients were not referred for genetic counselling

Since the sequencing was performed in collected specimens only, the mutations found were classified as somatic, germline homozygous, germline heterozygous, or variants of uncertain origin based on machine learning algorithms (ISOWN) followed by manual validation or, for indel variants, based on manual validation only (see Methodology).

**Fig. 2:**
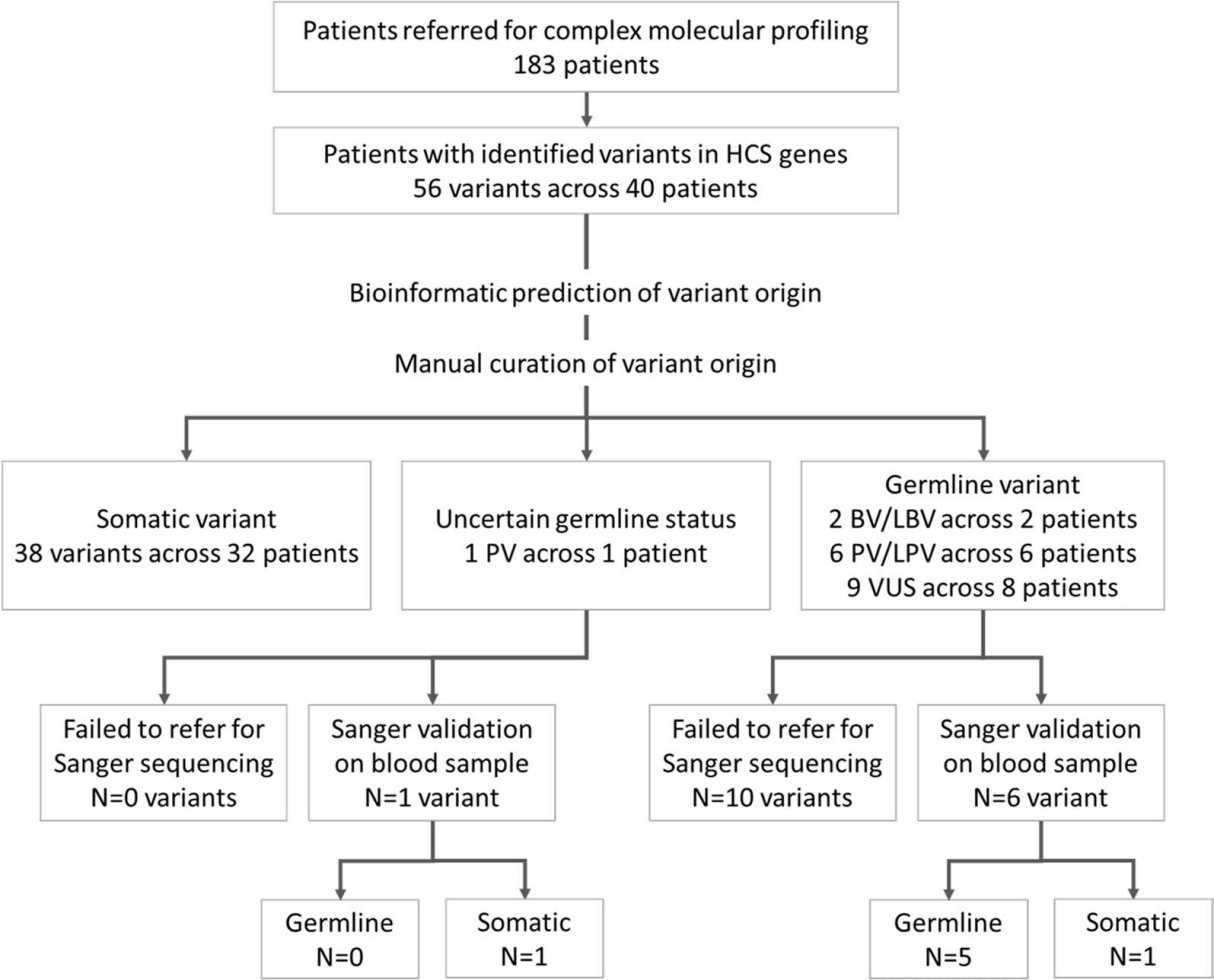
Study design and major results of variant detection and validation. PV: pathogenic variant, LPV: likely pathogenic, BV: benign, LBV: likely benign, VUS: variant of uncertain significance

Overall, ISOWN predictions were concordant with the results of Sanger-based validation for the 41 (97%) missense variants, including 10 germline and 31 somatic variants (Fig. 2). The most commonly mutated gene was *TP53*, which accounted for 48.2% of all the detected variants. All of the variants in *TP53* were somatic, based on the results of both ISOWN and Sanger-based validation. Mutations in DDR genes (*ATM, BLM, BRCA1, BRCA2, MLH1, MSH6, NBN, PMS2*) accounted for up to 40% of the variants. The majority of observed variants were detected in patients with colorectal (35.7% of all variants), gynecological (21.2%), and pancreatic (12.5%) cancers. A total of 38 variants across 32 patients were classified as somatic (Table 2, Fig. 2).

Following ACMG guidelines, clinical interpretation of germline variants or variants of uncertain origin was performed to classify them into pathogenic (PV), likely pathogenic (LPV), benign (BV), likely benign (LBV) variants, or variants of uncertain significance (VUS) [24]. In total, we detected 17 potentially-germline variants in 14 (8%) patients with various tumor types. Genetic counseling was recommended for all patients with PV/LPV/VUS variants of germline or uncertain origin. Germline variants classified as BV/LBV were not reported to the patients. Patients with the non-germline variants in genes associated with hereditary cancer syndrome (HCS) were not referred for genetic counseling. Five patients had both somatic and potentially-germline variants identified: 2 patients with colorectal, 1 patient with ovarian, 1 patient with stomach, and 1 patient with uterine cancers. In these patients, the somatic variants found were accompanied by at least one potentially-germline PV or VUS.

Sanger sequencing validation was performed in 7 patients with suspected germline variants. Of those, five variants (1 in ATM, 1 in APC, 1 in BLM, 1 in BRCA2, 1 in MSH6) were found to be germline, and two—in CDH1 and SMARCB2—were somatic. For the rest of the patients with PV/LPV/VUS variants of germline origin, Sanger sequencing was not performed due to one of the following reasons: (1) blood sample unavailable (2 cases); (2) patient preference (2 cases); (3) patient payor coverage circumstances (cost for Sanger sequencing was not included in the cost for complex molecular profiling) (8 cases).

In two cases, Sanger sequencing failed to detect potentially-germline or variants of uncertain origin in patients' blood samples. In particular, variants in SMARCB1 (HGVSp ESNT000151345:p.R154L) and CDH1 (HGVSp ENST00000261769:p.Y302X) genes were predicted to be somatic by ISOWN. After a thorough assessment of patients’ clinical characteristics and Sanger sequencing validation, these variants were labeled as likely germline (for SMARCB1) or of uncertain origin (for CDH1). In short, Sanger sequencing failed to detect these variants in the patients’ normal tissue, thus justifying their somatic origin.

In one case, Sanger sequencing validation following ISOWN prediction allowed for the capture of the origin of a misclassified variant. Specifically, VUS in the ATM gene (ENST00000278616:p.S1584R, VAF 49.8%) was detected in a patient with esophageal cancer and predicted to be somatic by ISOWN. Taking into account the clinical picture and the technical characteristics of the variant, we hypothesized that the variant may be, in fact, germline. Later, Sanger sequencing confirmed that the variant was germline, and the patient was referred for genetic counseling.

### Manual assessment of variant origin is beneficial over bioinformatics algorithms

**Fig. 3:**
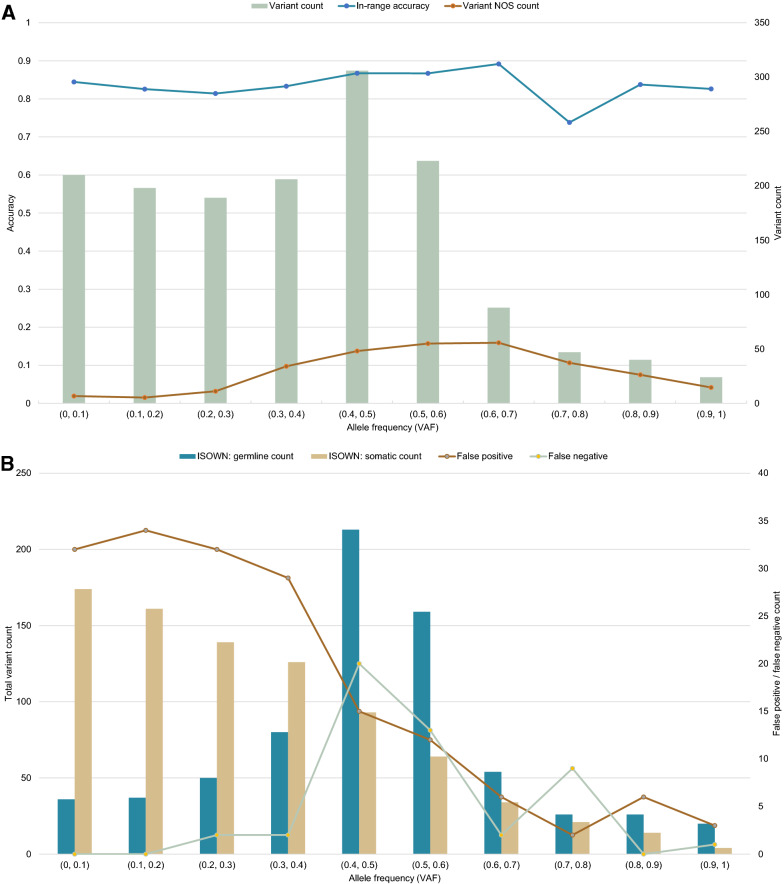

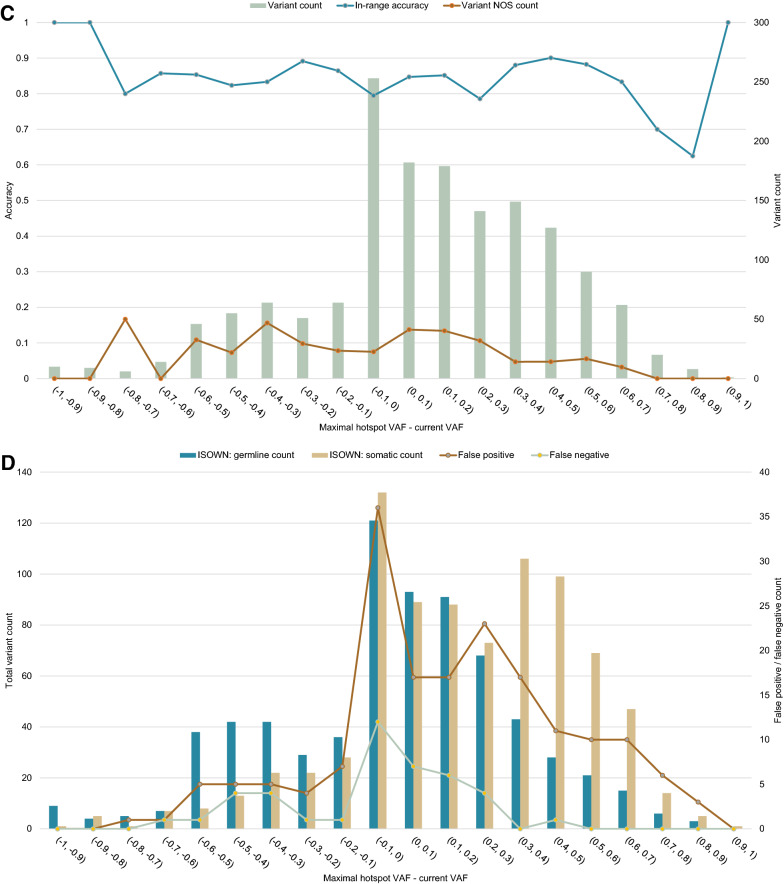
Retrospective analysis of variant origin prediction results provided by bioinformatics software (ISOWN). Manual curation was used as the gold standard. ISOWN accuracy does not depend on the variant allele frequency (NOS—variants with uncertain origin, as considered by manual curation) (**A**), in contrast to false-positive and false-negative rates (**B**). The same results were seen for different ranges of VAF distance between the studied variant and the known hotspot VAF in the same sample (**C**, **D**)

To assess the accuracy of tools for variant origin prediction, we manually assigned an origin (either germline, somatic or uncertain) to 1531 missense variants across 183 samples. Among them, 478 variants were found to be germline, 920 somatic, and 133 of uncertain origin (Fig. 3). Overall, ISOWN correctly predicted 436 (91%) of the variants as germline and 742 (80%) as somatic (Table 3). A subset of variants that were predicted to be somatic by ISOWN but classified as variants of uncertain origin based on manual validation had an average VAF of 48%, which differs significantly from that of all variants manually classified as somatic (28%, p-value < 0.001), as well as from all variants predicted to be somatic by ISOWN (29%, p-value < 0.001) (Table 4). VAFs were not significantly different between the set of variants considered as somatic based on manual classification and the set of variants predicted as being somatic based on ISOWN (mean 28% vs 29%, p-value 0.14). Next, we compared the difference between maximal VAF of hotspot variants (defined as the maximum VAF across variants within a single molecular profile satisfying the following criteria: COSMIC count of 100 and more; population frequency based on data from the TOPMED project of 0.001 and less) and VAF of all of the studied variants. These VAFs were different in the subgroup of variants of uncertain origin that ISOWN predicted to be somatic (mean—0.06%), compared to all of the detected somatic variants (21%, p-value < 0.001). This demonstrates how additional data, i.e. knowing the complete molecular profile of the patient, may be used in addition to the computationally predicted origin of individual variants.

**Table 3:** Accuracy of ISOWN predictions

Manual assignment	ISOWN	
	Germline	Somatic
Germline	TP = 436	FN = 42
Somatic	FP = 178	TN = 742
Variant of uncertain origin	87	46
Accuracy of ISOWN prediction: = 84.26% Sensitivity: = 91.21% Specificity: = 80.65% Precision: = 71.01%

**Table 4:** Variant annotation

	Manual assignment					ISOWN assignment	
	Germline	Somatic	Variants of uncertain origin			Germline	Somatic
			Total	ISOWN: germline	ISOWN: somatic		
Total	478	920	133	87	46	701	830
Annotated in COSMIC	151 (31.6%)	329 (35.8%)	34 (25.6%)	20 (23.0%)	14 (30.4%)	204 (29.1%)	310 (37.3%)
Annotated in dbSNP	423 (88.5%)	331 (36.0%)	82 (61.7%)	66 (75.9%)	16 (34.8%)	547 (78.0%)	289 (34.8%)
VAF (mean ± SD)	52% ± 15%	28% ± 21%	48% ± 16%	48% ± 17%	48% ± 14%	47% ± 20%	29% ± 20%
Minimal hotspot VAF (mean ± SD)	19% ± 19%	17% ± 19%	22% ± 18%	23% ± 17%	20% ± 18%	20% ± 20%	16% ± 17%
Maximal hotspot VAF (mean ± SD)	49% ± 29%	49% ± 28%	53% ± 27%	56% ± 27%	49% ± 27%	47% ± 30%	49% ± 28%
Maximal hotspot VAF—current (mean ± SD)	− 7.6% ± 32%	21% ± 31%	5.2% ± 28%	8.0% ± 28%	− 0.06% ± 26%	0.09% ± 34%	19% ± 31%
Maximal hotspot VAF—minimal hotspot VAF (mean ± SD)	26% ± 25%	31% ± 25%	31% ± 25%	33% ± 25%	28% ± 25%	27% ± 26%	32% ± 25%
EXAC frequency (mean)	1.28%	0.0017%	0.004%	0.005%	0.002%	0.88%	0.0013%
TOPMED frequency (mean)	1.23%	0.0012%	0.0026%	0.0029%	0.002%	0.84%	0.0013%
1000G frequency (mean)	1.16%	0.0015%	0.0038%	0.0037%	0.0039%	0.79%	0.0014%

ISOWN proved to be a useful tool for automated prediction of the variant origin; the overall sensitivity of ISOWN predictions was at 91.21%; the overall accuracy was at 84.26%; the precision or specificity was lower than that (Table 3). Notably, the accuracy of the predictions did not depend on variant allele frequency or the location of the variant concerning the hotspots (Fig. 2A, C). Across groups of variants with different VAFs, the accuracy was consistently higher than 70%. A comparison of the ISOWN predictions to the results of manual assignment of variant origin shows that ISOWN had more false-positive results than false-negatives. It is clear that the false-positive results were prevalent at low VAFs, and false-negative results peaked around a VAF of 50% (Fig. 2B). False-negative results were consistently found to be located close to the hotspots and had an allele frequency close to 50%. Other descriptors of false-negative variants did not significantly differ from descriptors of true somatic variants. A similar conclusion can be drawn for variants of uncertain significance that ISOWN classified as somatic (Table 4). Hence, our data suggest that ISOWN’s predictions of somatic variants are least reliable for variants with 50% VAF located in the vicinity of known hotspots.

### The general problem of somatic/germline variant discrimination across other projects

To estimate the risk of incorrect identification of the variant origin, somatic mutation data from the MSK-IMPACT cancer molecular epidemiology project were analyzed [28]. Of 58,337 unique somatic mutations identified in the MSK-IMPACT, a total of 14,102 (24%) were found in the dbSNP database (build 153). Of them, 1424 (2% of the total unique somatic mutations) were found in the 1000 Genomes Project [14] and 7012 (12% of the total unique somatic mutations) were found in the TOPMED project [16]. This indicates that between 12 and 24% of somatic variants may be located in the same genome positions as known germline variants. The frequency of germline variants may vary by ethnic background, making this an approximate estimation. Moreover, a total of 2188 (4%) and 608 (1%) somatic variants identified in the MSK-IMPACT project were previously annotated in the CLINVAR database as either pathogenic or likely pathogenic, respectively [29]. Of those, 45 variants were found in genes associated with HCS. These HSC variants were represented by a total of 82 occurrences across 78 (0.8%) different patients. For 11 (0.1% of patients, 95% CI 0.05–0.2%) of them, VAFs were in the range of 0.4 to 0.6. Within the tumor, normal pairs assessed in the frame of the MSK-IMPACT project, this estimation does account for germline variants that could be detected. Nevertheless, our data indicate that automated variant origin discrimination may lead to an incorrect assessment in 1% of patients, and Sanger validation may be required. In other words, normal tissue specimen Sanger sequencing should be recommended in case PV/LPV or VUS variant is identified in any of the HCS genes.

Next, we analyzed a total of 32, 10, and 1 tumor molecular profiling reports generated by FoundationOne®CDx, FoundationOne®Heme, and FoundationOne®Liquid companion diagnostic tests, respectively (Foundation Medicine, Inc.). We found that out of 187 variants reported across 43 reports, 100 (53%) were matched to an entry in the dbSNP database. In contrast, mutations annotated as somatic either by Sanger sequencing (N = 920) or by ISOWN (N = 830) were found in the dbSNP database with frequencies of only 36% and 35%, respectively. Moreover, in the MSK-IMPACT samples, only 24% of somatic variants were annotated in dbSNP. This may indicate a bias towards reporting germline variants in tumor-only sequencing datasets. Across FMI reported variants, a total of 76 (41%) and 32 (17%) were present in TOPMed and 1000Genomes population databases, respectively. Furthermore, a total of 44 (24%) variants had a population frequency of 0.1% and greater and a total of 10 (5%) variants had a population frequency of 1% and greater. Such statistics point towards a germline origin of these variants rather than a somatic origin, while the high population frequency of these variants indicates a possible lack of relevance to carcinogenesis and indicates potential problems with reporting germline variants across tumor profiling providers.

## Discussion

In oncology, NGS is predominantly used for the identification of somatic alterations. When found, these alterations guide therapeutic decisions on the applicability of the targeted therapies [30]. The majority of such alterations are either somatic mutations or fusions [28, 31]. Apart from somatic mutations, NGS is capable of identifying potentially germline variants, which may influence patient management as well as provide a rationale for timely genetic counseling and the implementation of screening the patients’ relatives [32]. However, while performing the sequencing in tumor specimens only, one should rely on either computationally predicting whether a certain variant is a germline one, or resort to a secondary study of normal tissues by Sanger sequencing. Additionally, variant origin analysis may eliminate the reporting of a fraction of irrelevant variants, such as potentially benign, likely-germline variants, or common-genetic polymorphisms.

It is expected that the discrimination between somatic and germline mutations will remain a crucial problem for the molecular profiling providers who use only tumor specimens as samples. In our study, we describe real-world outcomes of performing this type of sequencing for cancer patients. We describe the main considerations for classifying variants as somatic or likely germline using ISOWN and Sanger sequence validation, as well as highlight the importance of Sanger sequencing.

Though ISOWN can accurately predict the origin of up to 99% of missense variants [17], manual curation was only performed for all the controversial missense and non-missense variants. Both ISOWN and manual interpretation have their limitations. As discussed earlier, ISOWN can only predict the origin of missense variants and cannot be used to annotate indels. Other limitations of ISOWN, as mentioned in the original article, include decreased accuracy in cancer types with lower mutational load [17], such as breast cancer. Moreover, we show that ISOWN is the least accurate in predicting the origin of variants with VAF of around 50% or VAFs located close to hotspots. Considering all the limitations of ISOWN, manual curation remains an essential part of variant interpretation. We show that a combination of ISOWN and manual curation is effective in assigning either somatic or germline origin to the variants observed in the clinical setting.

Since NGS is an imperfect means for the detection of germline variants and ISOWN might misclassify potentially-germline variants, we propose that adding a category of “variants of uncertain origin” may be useful in the framework of manual validation of the variants to denote the changes which may not be unequivocally classified as somatic or germline. This approach aids in avoiding the misclassification of the origin of certain potentially-germline variants that otherwise would be classified as somatic. These variants should be further subjected to Sanger sequencing. In the case of the sequencing of the tumor samples, normal tissue Sanger sequencing may be required for at least 1% of cases referred for complex molecular profiling (95% CI 0.62–0.97%). In our real-world study, Sanger sequencing was required for 7 patients (3.8%, 95% CI 1.5–7.7%) with pathogenic or likely pathogenic potentially-germline or variants of uncertain origin. When coupled with the results of the MSK-IMPACT project, our data demonstrate that collecting patients’ normal tissue samples may be required for approximately 10% of real-world cases.

Apart from methodological obstacles in variant detection, the interpretation of sequencing variants as unequivocally germline or somatic remains challenging. Currently, several guidelines for the interpretation of the detected variants are available. The widely implemented ACMG guidelines [24], as well as their refined version, SHERLOCK [25], propose a 5-tier variant classification system. However, since these guidelines are focused on Mendelian hereditary conditions, their applicability to hereditary cancer syndromes is limited due to several reasons. First, patients with HCS may develop late-onset malignancies due to low expressivity of a trait [33], which precludes suspicion of its inherited nature. Secondly, the spectrum of malignancies among affected individuals in the same family may vary [34–37]. Monogenic hereditary cancer syndromes are rarely limited to the development of only one tumor type but rather associated with a range of malignancies. Coupled with variable penetrance and expressivity, both family history and variant segregation analysis may be used as strong or supporting evidence of pathogenicity or benign impact of a germline variant, as per ACMG guidelines, and complicate interpretation of the significance of a found variant.

**Fig. 4:**
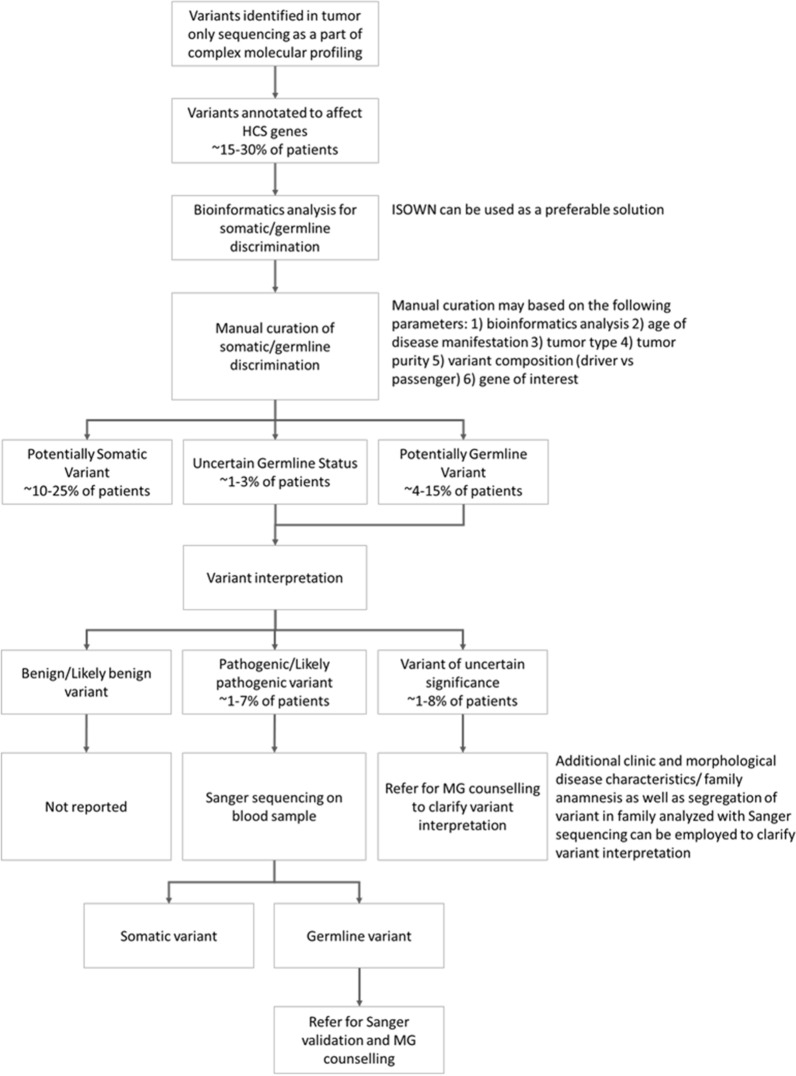
Proposed framework for managing patients with detected variants in Hereditary Cancer Syndrome (HCS) associated genes. MG: medical genetics

As a consequence, an assessment of all variants in genes associated with HCS within the framework of tumor-only complex molecular profiling should be considered. Based on real-world results, we provide methodological guidance for this kind of research (Fig. 4). Even as bioinformatic tools aid in distinguishing somatic vs germline origin by assessing variant allele frequency, presence of a variant in databases, nucleotide composition, CNV analysis, and more [38], misclassification events may occur, thus, warranting manual curation. Here we show that the errors may persist even after manual curation. Such errors may lead to incorrect management of patient and family counselling. Therefore, after completion of manual curation, normal tissue sequencing validation is required for all variants identified as potentially germline (including variants of uncertain origin). Moreover, patient management may depend on the potential pathogenicity of variants identified as germline or potentially germline. The significance of VUS may further be clarified based on clinical data, like morphological characteristics of the disease, family history, or segregation of the variant in the family. Therefore, following genetic counseling, further assessment may be recommended to refer patients with PV/LPV germline or potentially-germline variants for Sanger validation and/or family segregation analysis. Finally, BV or LBV should not be reported, as per ACMG guidelines [24].

In terms of management and screening procedures for patients with PV or LPV germline variants, current NCCN guidelines on Genetic/Familial High-Risk Assessment [11, 39] provide information only for a handful of genes relevant to a limited amount of tumor types. The development of such guidelines is complicated by the uncertainty of case–control studies and a lack of consensus on the appropriate threshold of hazard ratio (HR) for the selection of patients for the screening, as well as the spectrum of relevant tumor types. Additionally, there are currently no available guidelines on the management of patients with PV in other highly penetrant genes, such as BAP1. Moreover, no guidelines discuss the management of patients harboring PV in genes who had already been affected by cancer and had no knowledge of the genetic basis for the disease before genetic testing.

With the rare exception of well-characterized missense variants, the majority of annotated PVs are either frameshift or nonsense mutations [29]. Most of the detected missense variants are classified as VUS, as many of them have not been previously studied and their effects on protein function remain unknown. For instance, for the BRCA1 gene, only 4.5% of missense variants submitted to the ClinVar database are classified as PV, while 89% are classified as VUS [21]. While in silico algorithms are useful for effect prediction, they may only provide supporting evidence for defining pathogenicity [40].

In our study, 7 patients harbored potentially germline VUS in genes associated with HCS, suggesting an underlying inherited nature of their tumor. Whether the patients’ present diagnosis should be taken into account while interpreting potentially germline VUS should be further discussed by the scientific community. Efforts should be made to overcome methodological and clinical obstacles to the standardization of the genetic counseling of cancer patients referred for tumor molecular profiling.

The frequencies of incidental germline findings discovered during tumor molecular profiling were reported in many studies. In particular, Meric-Bernstam et al. showed that approximately 2.3% of patients with advanced cancer harbor previously unrecognised germline variants in genes associated with the development of HCS [41]. Some of these studies focus on specific tumor types. You et al. reported the overall frequency of pathogenic germline variants in patients with colorectal cancer of 9.9% [42]. In patients with lung cancer Tian et al. reported frequency of PV/LPV as 3.8% [43]. Another study of patients with advanced cancer revealed the occurrence of germline PV in HRD genes as 17.8% [44].

The discrepancy of the reported frequencies might be explained by several factors, such as differences of study designs, patient populations, as well as selected tumor types. Moreover, overarching analysis is precluded by some studies reporting PV and LPV only, while some others including VUS. In our study, we report real world frequencies of incidental germline variants detected in the course of routine tumor molecular profiling.

To sum up, in our study routine tumor molecular profiling revealed potentially-germline variants in 14 (8%) patients with various tumor types referred for tumor molecular profiling. While the prediction of the variant origins may be done by computational tools, manual curation of the tumor-only sequencing results is paramount. We suggest adding an additional category of “variants of uncertain origin”, which is of use when determining the origin of the sequencing variants. We highlight the importance of Sanger sequencing in patients’ normal tissue for validation of the origin of PV/LPV/VUS variants that are either potentially germline, or of uncertain origin. We also discuss the obstacles for the interpretation of variants that are potentially germline or of uncertain significance in cancer patients referred to tumor molecular profiling.

## Conclusions

Incidental findings of pathogenic germline variants are common in data from cancer patients referred for complex molecular profiling. We propose an algorithm for the management of variants in genes associated with HCS.

## Data Availability

Not applicable.

## Abbreviations

BV: Benign Variant
CNV: Copy Number Variation
FFPE: Formalin-Fixed Paraffin-Embedded
HCS: Hereditary Cancer Syndrome
LPV: Likely Pathogenic Variant
LBV: Likely Benign Variant
PV: Pathogenic Variant
SNV: Single-Nucleotide Variant
VAF: Variant Allele Frequency
VUS: Variant of Unknown Significance
